# Drug-induced social connection: both MDMA and methamphetamine increase feelings of connectedness during controlled dyadic conversations

**DOI:** 10.1038/s41598-023-43156-0

**Published:** 2023-09-22

**Authors:** Hanna Molla, Royce Lee, Sonja Lyubomirsky, Harriet de Wit

**Affiliations:** 1 Department of Psychiatry and Behavioral Neuroscience, University of Chicago, Chicago, IL USA; 2 Department of Psychology, University of California, Riverside, USA

**Keywords:** Human behaviour, Social behaviour

## Abstract

MDMA is a stimulant-like drug with distinctive empathogenic effects. Its pro-social effects, such as feelings of connectedness, may contribute to both its popularity as a recreational drug and its apparent value as an adjunct to psychotherapy. However, little is known about the behavioral processes by which MDMA affects social interactions. This investigation examined the effects of MDMA (100 mg versus placebo; *N* = 18) on feelings of connectedness with an unfamiliar partner during a semi-structured casual conversation. A separate study examined the effects of a prototypic stimulant methamphetamine (MA; 20 mg versus placebo; *N* = 19) to determine the pharmacological specificity of effects. Oxytocin levels were obtained in both studies. Compared to placebo, both MDMA and MA increased feelings of connection with the conversation partners. Both MDMA and MA increased oxytocin levels, but oxytocin levels were correlated with feeling closer to the partner only after MDMA. These findings demonstrate an important new dimension of the pro-social effects of MDMA, its ability to increase feelings of connectedness during casual conversations between two individuals. Surprisingly, MA had a similar effect. The findings extend our knowledge of the social effects of these drugs, and illustrate a sensitive method for assessing pro-social effects during in-person dyadic encounters.

## Introduction

The drug ± 3,4-Methylenedioxymethamphetamine (MDMA) is known to promote sociability and feelings of connection with others^1,2^. These effects are believed to contribute to its recreational use, and may also contribute to its effectiveness as an adjunct in the treatment of posttraumatic stress disorder^3^ by facilitating social processes during psychotherapy. However, researchers have a limited understanding of how psychoactive drugs affect social processes. Controlled laboratory studies can help to identify the mechanisms through which a drug alters social interactions. Until now, few controlled studies have examined the effects of MDMA on social interactions, the pharmacological specificity of such effects, or the underlying physiological mechanisms.

MDMA has been shown to produce prosocial effects in both laboratory animals and humans. In rodents, MDMA increases “adjacent lying” and social approach behaviors^4,5^. In humans, it increases subjective ratings of sociability as well as the amount of time participants spend interacting with others^6,7^. The drug also affects socio-emotional processing during computerized tasks in ways that are likely to impact social function^8–12^. Patients undergoing MDMA assisted therapy in group settings report that the drug intensifies interpersonal trust and empathy for others (see review^13^), and qualitative interviews^14^ of recreational MDMA users show that the drug is used not only at parties, but also at home with close friends. Yet, few studies have directly examined the effect of MDMA or other drugs on participants’ behavior and feelings during real-life interpersonal interactions^15^. One study comparing the subjective effects of MDMA in participants tested alone, with a research assistant, or with another participant^6^ found that MDMA produced slightly stronger effects when volunteers were with another participant who was also given MDMA. However, little is known about how the drug affects interpersonal connections during a social encounter.

An important question is whether the effects of MDMA are distinct from other stimulant drugs^1^. MDMA shares many receptor actions and behavioral effects with other amphetamines including increased synaptic levels of dopamine and norepinephrine^16^ and similar behavioral effects^17^ and pro-social effects in humans^1,18^. However, MDMA has more pronounced effects on serotonin receptors, which are thought to mediate its unique prosocial effects. Therefore, for comparison in this investigation, we also tested effects of the prototypic stimulant methamphetamine (MA) on feelings of connectedness.

It has been proposed that the prosocial effects of MDMA are mediated by its effects on oxytocin, a neuropeptide that promotes social bonding^19–21^. MDMA, but not methamphetamine, increases plasma levels of oxytocin, apparently through its actions on serotonin receptors^22,23^. Some studies^23^ have found that oxytocin levels in the blood after MDMA were positively correlated with subjective prosocial feelings, but others failed to observe this correlation^24,25^ . Amphetamine, in contrast, has little effect on oxytocin levels^21,26^. We obtained salivary levels of oxytocin in the present study to relate these to the drugs’ behavioral effects.

In this research study (NCT05123716), we examined the effects of single, moderate doses of MDMA (Study 1) and MA (Study 2) on feelings of closeness and connectedness while two individuals engaged in a conversation. We used a structured dyadic conversation task developed by Aron et al.^27^ to induce feelings of closeness and connectedness. Aron et al. (1997) showed that participants assigned to discuss “deep” topics reported stronger feelings of interpersonal connection compared to those assigned to discuss “shallow” (or small talk) topics. In the current study, participants engaged in the shallow conversation after administration of MDMA or MA. They conversed with a same-sex previously unknown partner in a 45-min semi-structured casual conversation, using topics that were relatively impersonal^27^. In both the MDMA and MA study, participants received drug on one session and placebo on the other session, and engaged in the conversation with a different partner during each session. They rated how connected and close they felt with their partners at the end of the sessions and again 1 week later. Saliva samples were obtained to assay oxytocin levels. We hypothesized that MDMA, but not MA, would increase feelings of connectedness to conversation partners and that this increase would be related to increased salivary oxytocin, relative to placebo.

## Methods and materials

### Studies 1 and 2

#### Study design

The two studies were conducted in separate groups of participants, one with MDMA (Study 1; *N* = 18) and one with MA (Study 2; *N* = 19). In both studies participants received drug (MDMA [100 mg] or MA [20 mg]) and placebo in randomized order during two in-lab sessions, under double-blind conditions. At the time of expected peak subjective drug effect 70–115 min after ingestion^8,10^, participants engaged in a semi-structured conversation with a novel, same-sex partner. Mood, cardiovascular and hormone levels were obtained during the sessions, and subjects rated their feelings of connectedness during the conversation and with the partner both after the session and one week later.

#### Participants

Participants were healthy male and female volunteers (age 18–35 years). They were recruited via posters and internet advertisements on social media, and underwent screening including a physical exam, electrocardiogram, psychiatric interview, medical history, and drug use history. Exclusion criteria were high blood pressure, abnormal EKG, any medical condition requiring regular medication, current DSM-V diagnosis of current substance dependence, mood, anxiety, or psychotic disorder, past treatment for drug or alcohol use disorders, or current pregnancy. Participants completed the Symptom Check List 90 Revised (SCL-90R)^28^ during screening. Inclusion criteria were BMI between 19 and 30, fluency in English, high school completion, and less than four alcoholic or caffeinated beverages a day. For Study 1 only, participants also had to report having used MDMA 1 to 40 times in their lifetime, with no history of adverse effects. They provided written, informed consent prior to beginning the study, which was approved by the Institutional Review Board of the Biological Sciences Division of the University of Chicago. All methods were performed in accordance with the declaration of Helsinki.

#### Drugs

For Study 1, MDMA in powdered form (100 mg; Organix Inc, MA) was placed in opaque size 00 capsules with lactose filler. For Study 2, MA tablets (5 mg, total dose 20 mg; Desoxyn, Lundbeck) were placed in an opaque size 00 capsule with dextrose filler, and placebo capsules contained only lactose or dextrose. The doses used here have been administered in previous studies without problems, and reliably produce subjective effects in healthy volunteers^9,29,30^.

### Procedure

#### Orientation

Participants attended a pre-study orientation session in which procedures were explained and informed consent was obtained. To minimize drug-specific expectancies, participants were informed that their capsules might contain a placebo, a stimulant such as amphetamine or MDMA, a sedative, or a hallucinogenic drug. They were instructed to fast for at least 8 h prior to the sessions, abstain from drug use for at least 2 days, cannabis use for at least 7 days, and alcohol use for 24 h before the sessions. They were informed that their recent drug use would be verified by urinalysis and breath alcohol testing before each session.

#### Study sessions

The two 4.5-h in-lab sessions were conducted from 9 am to 1:30 pm, separated by at least 4 days (Study 1) or 3 days (Study 2). Upon arrival in the laboratory at 9 am, drug abstention was verified by urinalysis (CLIAwaived Instant Drug Test Cup) and a breathalyzer (Alco-Sensor III, Intoximeters, St Louis, MO) for alcohol. Women were tested for pregnancy. Participants then completed pre-drug measures of subjective mood and provided baseline cardiovascular measures. They ingested a capsule at 9:30 am (Study 1: 100 mg MDMA or placebo; Study 2: 20 mg MA or placebo), and relaxed in a comfortable room for 1 h to allow the drug to be absorbed. At 10:40 am, they were taken to a separate room to engage in a 45-min conversation with a previously unknown, same-sex partner (see below). The partners were blind to the drug identity, and were trained to engage in natural social interactions. Throughout the session, at 30, 60, 120, 180, and 210 min after the capsule, subjective (Drug Effects Questionnaire [DEQ], Visual Analog Scale [VAS], and Profile of Mood States [POMS]) and cardiovascular measures were obtained. Single saliva samples for oxytocin level analysis were obtained at 120 min, when plasma levels of oxytocin are expected to peak after MDMA^10^. At 1:30 pm, participants completed questionnaires relating to the conversation, assessing partner closeness and connection. One week after the second session, the participants completed questionnaires rating their two conversations and conversation partners (i.e., from drug and placebo sessions).

### Subjective measures taken during study sessions

#### Drug effects questionnaire (DEQ)^31,32^

The DEQ consists of questions presented on a visual analog scale about the subjective effects of drugs. Participants are asked to rate on a 100 mm line, ranging from “Not at all” (0) to “Very much” (100), the extent to which they feel a drug effect, whether they like or dislike the drug effect, and if given a choice would they want to take more of the drug.

#### Visual analog scales (VAS)

The VAS consists of adjectives that describe MDMA or MA drug effects, including anxious, stimulated, insightful, sociable, confident, lonely, playful, loving, friendly, restless, trusting, appreciated, grateful, understood, and loved. Each adjective was rated from “Not at all” (0) to “Extremely” (100). The VAS and these adjectives have been used in previous MDMA and MA studies to assess subjective drug responses (see review^1^).

#### Profile of mood states (POMS)^33^

The POMS consists of 72 adjectives commonly used to describe momentary mood states. The POMS is sensitive to the effects of drugs in similar samples of healthy volunteers^34^. It consists of 8 subscales, whose scores can range from 0 to 60.

#### End of session questionnaire

At the end of each session, participants were asked to identify what drug they through they received: placebo, stimulant, sedative, or hallucinogen.

### Physiological measures taken during study sessions

#### Cardiovascular measures

Blood pressure and heart rate were monitored using portable blood pressure cuffs (Omron BP791IT, Omron Healthcare).

#### Hormonal measures

Saliva samples for oxytocin levels were collected 120 min after drug administration, after 1 h with no eating and 10 min with no drinking. They were immediately stored at − 80 °C. Oxytocin levels were measured using the Phoenix Pharmaceuticals oxytocin radioimmunoassay (Phoenix Pharmaceuticals, Burlingame CA; cat #RK-051–01). The samples were extracted by solid phase extraction prior to assay according to kit instructions. The limit of detection for this assay was 0.313 pg/mL and the intra and inter-assay coefficient of variance ranged from 4.06–5.85% and 7.89–12.8%, respectively.

### Ratings of the conversation

1. Conversation Questionnaire^35^. This questionnaire assesses the intensity of the connection between the partners. Responses ranged from “Not at all” (0) to “Extremely” (9). Participants completed 6 items from this questionnaire at the end of the sessions and again 1 week later during the follow-up. At follow-up they also completed partner ratings, how they perceived their partner would rate them on various characteristics, and how much they want to engage in another conversation with their partner.
2. Connection During Conversations Scale (CDCS)^36^. This 16-item questionnaire assesses connection experienced during an interpersonal interaction. Responses ranged from “Strongly disagree” (1) to “Strongly agree” (7). This measure has four subscales: shared reality (feeling a commonality), partner responsiveness (feeling understood and valued), participant interest (attentiveness), and affective experience (positivity). Participants completed these ratings at the end of the sessions.
3. Inclusion of Other in Self Scale (IOS)^37^. This single-item measure assesses the degree of connection felt with partners. It consists of 7 pairs of circles with varying degrees of overlap, ranging from no overlap (1; “no connection”) to most overlap (7; “felt extremely connected”). Participants completed these ratings at the end of the sessions and 1 week later during the follow-up.

### Conversation task during study sessions

During the 45 min conversations, participants were provided with “small talk” topics to discuss^27^, consisting of questions such as “What is your favorite holiday?” A different set of eight questions was provided every 15 min, and during the two sessions. Small talk topics were chosen to increase the potential to detect increases after drug administration, and minimize potential ceiling effects in connectedness ratings. Participants and their conversation partners were instructed to engage in a natural conversation, using the topics provided to them as prompts. The participants could choose not to discuss any question if either of them preferred not to. Their conversations were audiotaped (not reported here).

### Data analysis

Studies 1 and 2 were analyzed separately. The primary outcome measures were the three conversation rating questionnaires completed at the end of each session. Each question was analyzed separately because individual questions addressed different aspects of the interaction, and it was not known how drugs would affect each question. Mixed model analysis of variance (ANOVA) was conducted using drug condition as a within-subject variable, and sex and condition order (drug-placebo, placebo-drug) as between subject factors (SPSS Version 25), for each item. Sex and drug order were included as factors because the influence of sex and test–retest reliability on this task were not known. For subjective and cardiovascular measures taken repeatedly throughout each session, we calculated the peak change from baseline (pre-drug) on each session and compared drug vs. placebo scores using two-tailed paired *t*-tests (SPSS Version 25) within each study. Pearson correlations (SPSS Version 25) were conducted to determine the relationship between oxytocin levels and closeness ratings. The criterion for significance was p < 0.05.

## Results

Two participants were excluded from analysis of Study 1, one because he expressed strong negative feelings about a conversation partner and another because he socialized with his study partner following the in-lab sessions (before the follow-up).

### Study demographics

Most participants in both studies were in their twenties, had at least some college, and reported low-to-moderate prior drug use. The groups did not differ in BMI and current drug use (Table 1), but participants in Study 1 reported higher lifetime drug use, consistent with the inclusion criterion of having used MDMA at least 1 time (see “Methods”).

**Table 1:** Participant demographics.

Participant demographics	Study 1 (MDMA)	Study 2 (MA)
	*n* or mean (SD)	*n* or mean (SD)
Sex		
M/F	11/6	8/11
Ethnicity		
African American	0	2
Asian	2	3
Caucasian	9	14
Other	6	0
Age	26.6 (3.2)	22.1 (3.0)
BMI	22.4 (2.7)	22.3 (3.0)
Higher education in years	4.0 (1)	3.0 (1.2)
Current drug use		
Caffeinated drinks per day	1.6 (1.0)	0.9 (1.1)
Cigarettes per day	0.4 (1.0)	0.06 (0.2)
Alcoholic drinks per week	2.6 (2.0)	2.1 (1.7)
Cannabis use in past 30 days	4.9 (8.2)	2.1 (3.7)
Lifetime drug use		
Sedatives	0.2 (0.4)	0 (0)
Stimulants (other than MDMA)	11.82 (15.5)	0.3 (1.2)
Opiates	0.1 (0.3)	0.1 (0.2)
Psychedelics	11.2 (8.5)	1.6 (3.0)
MDMA	6.7 (5.0)	0.3 (0.7)
Median and (range) lifetime use of MDMA	5 (1–20)	0 (0–3)
Median and (range) lifetime use of stimulants	3 (0–50)	0 (0–5)

### Closeness and connection measures (end of session)

#### Study 1 (MDMA)

There were no significant interactions between drug condition and sex or session order for any of the connectedness measures.

**Figure 1:**
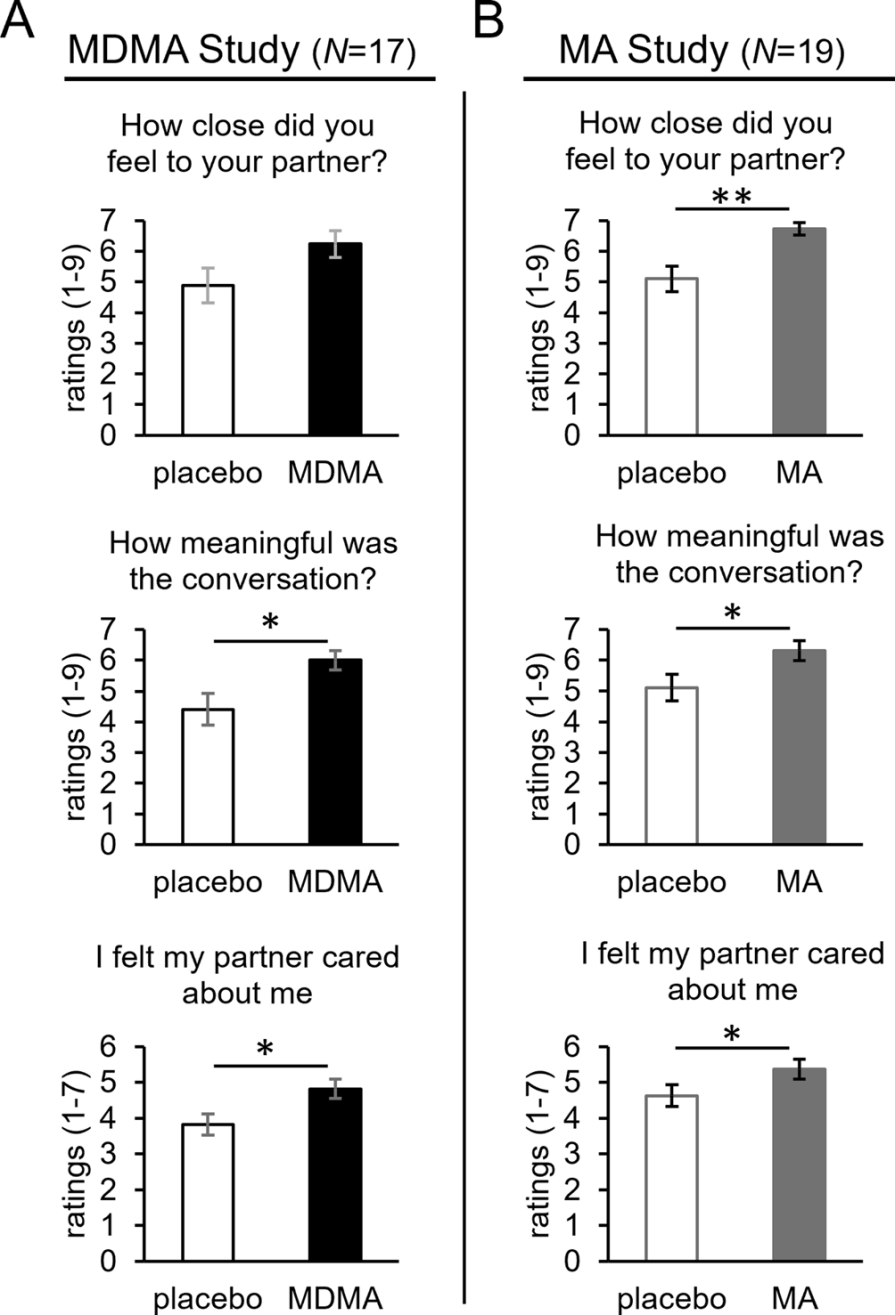
Participant ratings on three items from the Conversation Questionnaire and CDCS for the MDMA study (**A**), and the methamphetamine study (**B**). Bars depict mean ratings ± SEM. Significance between drug vs. placebo, *p < 0.05, ** p < 0.005, *** p < 0.0005.

On the Conversation Questionnaire taken at the end of each session, relative to placebo, MDMA significantly increased ratings of liking the conversation partner (*F*_1,13_ = 8.2, *p* = 0.01, ƞ_p_^2^ = 0.39) and finding the conversation more enjoyable (*F*_1,13_ = 7.1, *p* = 0.02, ƞ_p_^2^ = 0.36) and meaningful (*F*_1,13_ = 8.9, *p* = 0.01, ƞ_p_^2^ = 0.41; see Fig. 1A and Table 2).

**Table 2:** Conversation questionnaire and IOS ratings of conversation partners at the end of each in-lab session for Studies 1 [MDMA] and 2 [MA].

Conversation questionnaire and IOS	MDMA Study (*N* = 17)			MA Study (*N* = 19)		
	PLAC Mean ± SEM	MDMA Mean ± SEM	*P*	PLAC Mean ± SEM	MA Mean ± SEM	*P*
How enjoyable did you find the conversation?	6.2 ± 0.5	7.6 ± 0.3	0.02*	6.2 ± 0.4	7.9 ± 0.2	0.002**
How meaningful did you find the conversation?	4.2 ± 0.6	6.0 ± 0.4	0.01*	5.1 ± 0.4	6.3 ± 0.4	0.02*
How much did you like your conversation partner?	6.2 ± 0.6	8.1 ± 0.4	0.01*	6.7 ± 0.4	8.1 ± 0.2	0.001**
How much did you think your conversation partner liked you?	5.9 ± 0.5	6.9 ± 0.4	0.2	6.0 ± 0.4	7.0 ± 0.2	0.009*
How much did you and your partner have in common with one another?	5.7 ± 0.6	6.3 ± 0.4	0.4	5.8 ± 0.3	6.1 ± 0.4	0.6
How close did you feel to your conversation partner?	4.9 ± 0.6	6.4 ± 0.5	0.08	5.1 ± 0.4	6.7 ± 0.2	0.005**
Inclusion of other in self scale (IOS)	3.0 ± 0.4	4.0 ± 0.3	0.05	3.9 ± 0.2	4.9 ± 0.3	0.01*

On the IOS scale, MDMA elicited a trend of greater connection toward their partner, compared to placebo (*F*_1,13_ = 4.7, *p* = 0.05, ƞ_p_^2^ = 0.27; see Table 2).

On the CDCS, MDMA significantly increased ratings on 5 of the statements describing their partners: “They were interested in my thoughts and feelings,” (*F*_1,13_ = 7.7, *p* = 0.02, ƞ_p_^2^ = 0.37), “They really understood who I am,” (*F*_1,13_ = 5.1, *p* = 0.04, ƞ_p_^2^ = 0.28), “I felt they cared about me,” (*F*_1,13_ = 8.9, *p* = 0.01, ƞ_p_^2^ = 0.41), “They respected my beliefs and opinions,” (*F*_1,13_ = 5.4, *p* = 0.04, ƞ_p_^2^ = 0.29), and “I was interested in their thoughts and feelings,” (*F*_1,13_ = 6.1, *p* = 0.03, ƞ_p_^2^ = 0.32; see Table 3). We also assessed subscale scores and MDMA elicited greater ratings on partner responsiveness, (*F*_1,13_ = 15.4, *p* = 0.002, ƞ_p_^2^ = 0.54), and participant interest, (*F*_1,13_ = 5.4, *p* = 0.04, ƞ_p_^2^ = 0.30), relative to placebo (see Supplementary Table 1).

**Table 3:** CDCS item ratings of conversation partners at the end of each in-lab session for Studies 1 [MDMA] and 2 [MA].

Connection during conversations scale	MDMA Study (*N* = 17)			MA Study (*N* = 19)		
	PLAC Mean ± SEM	MDMA Mean ± SEM	*P*	PLAC Mean ± SEM	MA Mean ± SEM	*P*
I felt “in sync” with them	5.1 ± 0.4	6.0 ± 0.2	0.1	4.7 ± 0.3	6.0 ± 0.2	0.003**
I felt like we shared a lot in common	5.4 ± 0.4	5.7 ± 0.3	0.6	5.0 ± 0.3	5.8 ± 0.3	0.06
I felt that we saw the world in the same way	4.9 ± 0.5	5.4 ± 0.4	0.3	4.9 ± 0.3	5.6 ± 0.3	0.09
They were able to relate to my experiences	5.2 ± 0.4	5.5 ± 0.4	0.7	5.5 ± 0.2	5.9 ± 0.3	0.3
They were interested in my thoughts and feelings	5.5 ± 0.4	6.4 ± 0.2	0.02*	5.6 ± 0.2	6.1 ± 0.2	0.07
They really understood who I am	3.5 ± 0.4	4.2 ± 0.3	0.04*	4.4 ± 0.3	5.0 ± 0.3	0.2
I felt that they cared about me	3.9 ± 0.3	4.9 ± 0.3	0.01*	4.6 ± 0.3	5.3 ± 0.2	0.02*
They respected my beliefs and opinions	5.4 ± 0.3	6.5 ± 0.2	0.04*	6.4 ± 0.2	6.6 ± 0.1	0.1
I was interested in their thoughts and feelings	5.2 ± 0.4	6.4 ± 0.2	0.03*	5.6 ± 0.3	6.8 ± 0.1	0.005*
I was truly attentive during the interaction	5.1 ± 0.3	5.6 ± 0.4	0.4	5.7 ± 0.3	6.5 ± 0.2	0.045*
I thought they were boring	3.0 ± 0.5	1.6 ± 0.3	0.03*	2.5 ± 0.3	1.2 ± 0.1	0.002**
I was distracted during the conversation	2.9 ± 0.4	3.3 ± 0.5	0.5	2.3 ± 0.3	2.0 ± 0.3	0.5
I felt that it was hard to communicate with them	2.3 ± 0.3	2.1 ± 0.3	0.7	2.7 ± 0.3	1.3 ± 0.1	0.001**
I couldn’t wait for the interaction to end	2.2 ± 0.4	1.6 ± 0.3	0.3	2.8 ± 0.4	1.1 ± 0.1	0.001**
I felt that my energy was drained by the interaction	2.6 ± 0.4	2.7 ± 0.3	0.9	2.8 ± 0.4	1.7 ± 0.3	0.03*
I was nervous during the interaction	2.4 ± 0.4	3.1 ± 0.5	0.1	2.8 ± 0.4	2.5 ± 0.4	0.4

#### Study 2 (MA)

There were no significant main effects of sex or order, but there were isolated interactions between these variables and drug. No systematic patterns were detected in these interactions.

On the Conversation Questionnaire, MA significantly increased participants’ ratings of how enjoyable (*F*_1,15_ = 13.8, *p* = 0.002, ƞ_p_^2^ = 0.48) and meaningful (*F*_1,15_ = 6.3, *p* = 0.02, ƞ_p_^2^ = 0.30) they found the conversation, how much they liked their conversation partner (main effect of drug, *F*_1,15_ = 15.2, *p* = 0.001, ƞ_p_^2^ = 0.50; drug x order interaction, *F*_1,15_ = 8.4, *p* = 0.01, ƞ_p_^2^ = 0.36), how much they thought their conversation partner liked them (*F*_1,15_ = 9.1, *p* = 0.009, ƞ_p_^2^ = 0.38; drug x order interaction, *F*_1,15_ = 5.3, *p* = 0.04, ƞ_p_^2^ = 0.26), and how close they felt to their partner (*F*_1,15_ = 10.9, *p* = 0.005, ƞ_p_^2^ = 0.42; see Fig. 1B and Table 2).

MA significantly increased feelings of partner connection, compared to placebo, on the IOS (*F*_1,15_ = 8.6, *p* = 0.01, ƞ_p_^2^ = 0.36; see Table 2).

On the CDCS, MA resulted in higher ratings for 4 of the statements: “I felt in ‘sync’ with [my partner],” (main effect of drug, *F*_1,15_ = 9.1, *p* = 0.009, ƞ_p_^2^ = 0.38; drug x order interaction, *F*_1,15_ = 5.1, *p* = 0.04, ƞ_p_^2^ = 0.26), “I felt that they cared about me,” (*F*_1,15_ = 6.8, *p* = 0.02, ƞ_p_^2^ = 0.31), “I was interested in their thoughts and feelings,” (*F*_1,15_ = 10.7, *p* = 0.005, ƞ_p_^2^ = 0.42), and “I was truly attentive during the interaction.” (*F*_1,15_ = 4.8, *p* = 0.045, ƞ_p_^2^ = 0.24). Relative to placebo, MA also significantly decreased conversation ratings for 4 of the statements—namely, “I thought they were boring,” (*F*_1,15_ = 14.7, *p* = 0.002, ƞ_p_^2^ = 0.49), “I felt that it was hard to communicate with them,” (main effect of drug, *F*_1,15_ = 17.3, *p* = 0.001, ƞ_p_^2^ = 0.54; drug x sex interaction; drug x order interaction, *F*_1,15_ = 9.3, *p* = 0.008, ƞ_p_^2^ = 0.38), “I couldn’t wait for the interaction to end,” (*F*_1,15_ = 19.4, *p* = 0.001, ƞ_p_^2^ = 0.57), and “I felt that my energy was drained by the interaction” (*F*_1,15_ = 5.6, *p* = 0.03, ƞ_p_^2^ = 0.27; see Table 3). Additionally, MA increased scores on all four subscales of the CDCS: shared reality, (main effect of drug, *F*_1,15_ = 7.2, *p* = 0.02, ƞ_p_^2^ = 0.32; drug x order interaction,* F*_1,15_ = 8.3, *p* = 0.01, ƞ_p_^2^ = 0.36), partner responsiveness, (*F*_1,15_ = 6.7, *p* = 0.02, ƞ_p_^2^ = 0.31), participant interest, (main effect of drug, *F*_1,15_ = 17.2, *p* = 0.001, ƞ_p_^2^ = 0.53; drug x order interaction,* F*_1,15_ = 5.0, *p* = 0.04, ƞ_p_^2^ = 0.25), and affective experience, (main effect of drug, *F*_1,15_ = 18.5, *p* = 0.001, ƞ_p_^2^ = 0.55; drug x order interaction,* F*_1,15_ = 6.8, *p* = 0.02, ƞ_p_^2^ = 0.31; see Supplementary Table 1).

### Direct subjective drug effects

DEQ: Both MDMA (Study 1) and MA (Study 2) significantly increased ratings of “feel drug,” “like drug,” “dislike drug,” “high,” and “want more” (Supplementary Table 2).

VAS: MDMA (Study 1) and MA (Study 2) significantly increased VAS ratings compared to placebo on stimulated, insightful, sociable, loving, and friendly. Only MDMA increased ratings for trusting, appreciated, grateful, and loved, while only MA increased ratings for understood, compared to placebo (Supplementary Table 2).

POMS: MDMA (Study 1) and MA (Study 2) increased scores on elation, friendliness, and vigor. Only MDMA increased ratings for anxiety and confusion, while only MA significantly decreased fatigue ratings relative to placebo (see Supplementary Table 2).

On the end of session questionnaire, in Study 1, 34 percent of participants correctly identified placebo and 75 percent correctly identified MDMA as a stimulant. For Study 2, 74 percent of participants correctly identified placebo, and 74 percent correctly identified MA as a stimulant.

### Closeness and connection measures (follow-up)

During the online follow-up 1 week later, participants completed questionnaires regarding their two conversation partners.

#### Enjoyment and meaningfulness of the two conversations

In Study 1, participants reported finding the conversation after MDMA to be more meaningful than the conversation after placebo (*F*_1,12_ = 5.8, *p* = 0.03, ƞ_p_^2^ = 0.33). In Study 2, participants reported the conversation after MA to be more enjoyable (main effect of drug, *F*_1,15_ = 22.6, *p* = 0.0003, ƞ_p_^2^ = 0.60; drug x order, *F*_1,15_ = 5.7, *p* = 0.03, ƞ_p_^2^ = 0.27) and meaningful (*F*_1,12_ = 12.9, *p* = 0.003, ƞ_p_^2^ = 0.46) than after placebo, and reported greater liking (main effect of drug, *F*_1,15_ = 8.6, *p* = 0.01, ƞ_p_^2^ = 0.36; drug x order, *F*_1,15_ = 8.6, *p* = 0.01, ƞ_p_^2^ = 0.36) and feeling close (*F*_1,12_ = 12.0, *p* = 0.003, ƞ_p_^2^ = 0.44) to their partner, and having more in common with the partner (main effect of drug, *F*_1,15_ = 6.9, *p* = 0.02, ƞ_p_^2^ = 0.32; drug x order x sex, *F*_1,15_ = 4.7, *p* = 0.046, ƞ_p_^2^ = 0.24; see Table 4).

**Table 4:** Partner ratings on closeness measures during online follow-up one week after the last in-lab session for Studies 1 [MDMA] and 2 [MA].

Conversation questionnaire and IOS	MDMA study (*N* = 16)			MA study (*N* = 19)		
	PLAC Mean ± SEM	MDMA Mean ± SEM	*P*	PLAC Mean ± SEM	MA Mean ± SEM	*P*
How enjoyable did you find the conversation?	5.7 ± 0.7	6.7 ± 0.4	0.2	5.7 ± 0.4	7.7 ± 0.2	0.0003***
How meaningful did you find the conversation?	4.6 ± 0.7	6.2 ± 0.5	0.03*	4.9 ± 0.4	6.5 ± 0.4	0.003**
How much did you like your conversation partner?	6.0 ± 0.6	7.3 ± 0.4	0.08	6.7 ± 0.4	7.8 ± 0.3	0.01*
How much did you think your conversation partner liked you?	5.7 ± 0.4	6.2 ± 0.4	0.4	5.9 ± 0.5	6.8 ± 0.3	0.06
How close did you feel to your conversation partner?	4.9 ± 0.7	6.1 ± 0.5	0.1	4.8 ± 0.4	6.6 ± 0.3	0.003**
How much did you and your partner have in common with one another?	5.1 ± 0.6	6.1 ± 0.4	0.2	5.2 ± 0.3	6.6 ± 0.5	0.02*
Evaluate your partner on the following characteristics						
Intelligent	6.9 ± 0.5	7.0 ± 0.2	0.7	7.4 ± 0.2	8.0 ± 0.2	0.08
Competent	6.9 ± 0.4	7.3 ± 0.3	0.3	7.5 ± 0.3	8.1 ± 0.2	0.1
Kind	7.5 ± 0.3	7.8 ± 0.3	0.4	7.5 ± 0.4	8.2 ± 0.2	0.09
Physically attractive	5.5 ± 0.4	6.2 ± 0.4	0.04*	5.5 ± 0.4	6.1 ± 0.4	0.07
Friendly	7.2 ± 0.4	7.8 ± 0.3	0.1	7.3 ± 0.3	7.7 ± 0.2	0.2
Warm	6.5 ± 0.4	7.6 ± 0.3	0.04*	6.7 ± 0.4	7.7 ± 0.2	0.03*
How do you think your partner would evaluate you on the following characteristics						
Intelligent	6.8 ± 0.1	6.4 ± 0.2	0.07	6.6 ± 0.3	7.1 ± 0.2	0.006*
Competent	6.9 ± 0.3	6.1 ± 0.3	0.008*	6.8 ± 0.3	7.0 ± 0.3	0.2
Kind	6.7 ± 0.3	7.2 ± 0.3	0.1	7.3 ± 0.3	7.6 ± 0.3	0.04*
Physically attractive	5.7 ± 0.3	5.8 ± 0.3	0.6	5.8 ± 0.4	5.8 ± 0.4	0.9
Friendly	6.6 ± 0.3	7.3 ± 0.2	0.07	7.1 ± 0.3	7.4 ± 0.2	0.1
Warm	6.1 ± 0.3	7.3 ± 0.3	0.009*	6.9 ± 0.3	7.4 ± 0.2	0.002**
Imagine the next 7 days of your life. If you had the option, how many of those days would you like to have another conversation with your partner?	2.2 ± 0.4	2.6 ± 0.4	0.3	2.2 ± 0.2	3.4 ± 0.4	0.009*
Inclusion of other in self scale (IOS)	3.1 ± 0.4	3.4 ± 0.4	0.6	3.6 ± 0.3	4.0 ± 0.4	0.2

#### Ratings of partners after drug versus placebo

In Study 1, participants rated their MDMA partners as being significantly more physically attractive (*F*_1,12_ = 5.5, *p* = 0.04, ƞ_p_^2^ = 0.31),and warm (drug, *F*_1,12_ = 5.1, *p* = 0.04, ƞ_p_^2^ = 0.30; drug x order interaction, *F*_1,12_ = 7.9, *p* = 0.02, ƞ_p_^2^ = 0.40) compared to their placebo partners. In Study 2, participants rated their MA partners significantly higher on warmth, (*F*_1,15_ = 5.5, *p* = 0.04, ƞ_p_^2^ = 0.31) compared to their placebo partner (see Table 4).

#### Perceptions of the two partners’ experiences

Participants in Study 1 indicated that their partners during the MDMA session perceived them as being less competent (drug, *F*_1,12_ = 10.0, *p* = 0.008, ƞ_p_^2^ = 0.45; drug x sex interaction,* F*_1,12_ = 14.6, *p* = 0.002, ƞ_p_^2^ = 0.55; drug x order interaction, *F*_1,12_ = 17.3, *p* = 0.001, ƞ_p_^2^ = 0.59) and more warm (*F*_1,12_ = 9.6, *p* = 0.009, ƞ_p_^2^ = 0.44) than their placebo partners. In Study 2 participants indicated that their partners during the MA session perceived them as being more intelligent (*F*_1,15_ = 10.0, *p* = 0.006, ƞ_p_^2^ = 0.40), kind (*F*_1,15_ = 5.2, *p* = 0.04, ƞ_p_^2^ = 0.26), and warm (drug, *F*_1,15_ = 13.8, *p* = 0.002, ƞ_p_^2^ = 0.48; drug x sex x order, *F*_1,15_ = 8.0, *p* = 0.01, ƞ_p_^2^ = 0.35), than their placebo partners (see Table 4).

#### Wanting another conversation

When participants were asked how many days out of the next 7 days they would like to have another conversation with their conversation partner, no significant differences emerged between the placebo and MDMA partners in Study 1. In Study 2, participants rated wanting to spend significantly more time with their MA conversation partners compared to their placebo conversation partners (*F*_1,15_ = 9.0, *p* = 0.009, ƞ_p_^2^ = 0.37; see Table 4).

#### Closeness with other

At follow-up, neither drug (Studies 1 and 2) significantly increased ratings of closeness on the IOS with their partners (see Table 4).

### Physiological measures

#### Studies 1 and 2 (MDMA; MA)

**Figure 2:**
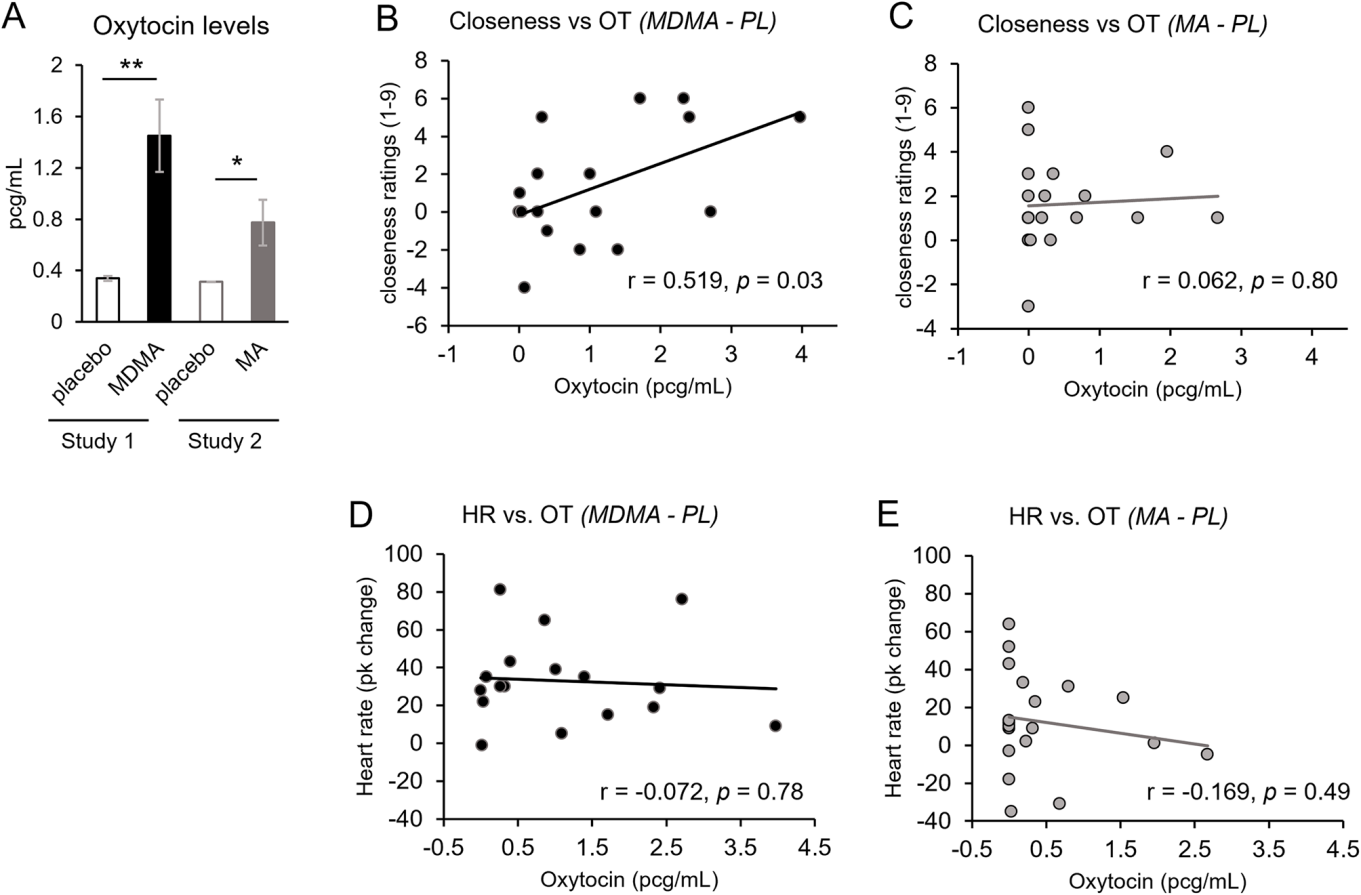
(**A**) Salivary oxytocin levels during Study 1’s placebo session (white bar) and MDMA session (black bar), and Study 2’s placebo session (white bar) and MA session (gray bar). Bars depict mean levels ± SEM. Significance between drug vs. placebo, *p < 0.05, ** p < 0.005. Top row scatter plots represent the relationship between closeness ratings, using the Conversation Questionnaire item: ‘*how close did you feel to your partner?*’, and salivary oxytocin (OT) levels, after MDMA (**B**), and after methamphetamine (**C**). Bottom row scatter plots represent the relationship between peak change heart rate, and salivary oxytocin (OT) levels after MDMA (**D**), and after methamphetamine (**E**). All scatter plot values were calculated by subtracting each participant’s placebo session from their drug session.

Both drugs significantly increased systolic blood pressure, diastolic blood pressure, and heart rate, relative to placebo (see Supplementary Table 4). Both drugs also significantly increased mean salivary oxytocin levels relative to placebo (see Fig. 2A and Supplementary Table 3). However, salivary oxytocin levels were below detectable limits in a large proportion of samples in both studies. In Study 1, 12 out of 17 samples from placebo sessions were below detectable limits, and 1 out of 17 samples after MDMA. In Study 2, 18 out of 19 placebo samples and 9 out of 19 MA samples were below detectable limits. These samples were recorded as the lower assay limit, 0.313 pg/mL.

### Oxytocin correlations

Next, we examined the relationship between salivary oxytocin (drug minus placebo) and feelings of closeness, using ratings (drug minus placebo) from the item “How close did you feel to your partner?” on the Conversation Questionnaire. Oxytocin levels were positively correlated with closeness ratings in the MDMA study (Study 1; *p* = 0.03; see Fig. 2B) but not in the MA study (Study 2; *p* = 0.8; see Fig. 2C). To determine the specificity of oxytocin’s effect, we also examined the relationship between oxytocin and a cardiovascular measure, heart rate. We found no significant correlations between oxytocin and heart rate with either MDMA (Study 1; see Fig. 2D) or MA (Study 2; see Fig. 2E).

## Discussion

As hypothesized, MDMA led to a robust increase in feelings of connection to conversation partners relative to placebo. Participants reported that they liked their partners more, and the conversations were more meaningful after MDMA. Interestingly, and contrary to our expectations, feelings of connectedness and enjoyment of the conversation were also increased by MA. Unfortunately, many of the oxytocin levels were below detectable limits, making it difficult to draw conclusions about these results. Nevertheless, MDMA and, to a lesser extent, MA increased salivary oxytocin levels, and oxytocin levels after MDMA (but not MA) were positively related to ratings of closeness with the partner. Taken together, these findings illustrate a novel method for assessing the effects of drugs on social connection, and yield the surprising finding that both MDMA and MA produce strong feelings of connectedness with a stranger after a brief conversation. The increased feelings of closeness and connectedness during a social interaction may contribute to the drugs’ nonmedical use, and may also contribute to the effect of MDMA as an adjunct to psychotherapy.

In Study 1, MDMA increased participants’ ratings of liking their partners, feeling connected and finding the conversation enjoyable and meaningful. This finding extends previous anecdotal reports, and some laboratory studies, reporting pro-social effects of MDMA on behavioral tasks with a social component. In prior studies, MDMA enhanced behavioral responses to emotional faces, increased subjective feelings of sociability and friendliness^7,38,39^ and produced feelings of closeness, trust, and openness^12^. However, no previous studies have examined the direct effect of MDMA on social connectedness in an in-person, socially relevant context, under double blind conditions. Our results show that the drug enhances the perceived quality and depth of a real-life social encounter.

The behavioral and neural mechanisms by which MDMA enhances social interactions are not known. The drug may alter processing of emotional states of others, such as the ability to recognize negative emotional facial expressions and the neural responses to negative stimuli^7–9^. MDMA also enhances ventral striatal responses to positive facial expressions^7^ and increases explicit and implicit emotional empathy toward others^9^. These MDMA induced alterations in socioemotional processing may create a more positive environment during interpersonal interactions, resulting in a more rewarding interaction and enhanced feelings of connection.

Surprisingly, we found similar increases in ratings of closeness and connection to conversation partners, as well as enjoyment of the conversation after MA. This finding was contrary to our expectation that feelings of connectedness during an interpersonal encounter would be specific to MDMA. Although stimulants such as d-amphetamine and methamphetamine have been found to increase self-ratings of feeling social and talkative^40^, to increase the amount of talking, and to improve ability to detect emotions in others^18^, prototypic stimulants are not typically thought to promote feelings of closeness and connection. Until now, no studies have examined this social effect of drugs, and the present findings suggest that MA may share this effect with MDMA. Indeed, this finding raises questions about the nature of closeness and connection. For example, the commonalities between MDMA and MA on increased feelings of closeness raise the possibility that feelings of connectedness are related to the quantity or the quality of verbal interaction, rather than an underlying emotional connectedness. That is, increased verbal communication may indirectly facilitate interaction and even personal disclosure during a conversation.

In the present study, the enhanced meaningfulness of the conversation induced by MDMA and MA were still present at least one week after the conversations. This suggests that interpersonal events experienced under the influence of a drug could have lasting effects on social interactions. Indeed, although we were unable to compare the two drugs directly, the lasting effects appeared to be more pronounced after MA compared to after MDMA. Whether this reflects true differences between the drugs will be determined in future studies.

The present findings with MDMA and MA can be compared to a recent study conducted in our laboratory^41^, examining feelings of connectedness after shallow conversations (such as that used here) and deep conversations, as designed by Aron et al.^27^ In the deep conversations, participants discussed topics that were more personally significant. In that study, in which no drugs were administered, the deep conversation increased reports of feeling connected relative to the shallow conversations. Interestingly, the increased reports of feeling connected after the deep conversations were comparable in magnitude to the increased reports of feeling connected after MDMA and MA in the present report. It will be of interest in the future to determine what aspects of the conversations are associated with feelings of connection.

The results with oxytocin are difficult to interpret for several reasons. First, we obtained just a single sample on each session, and no sample before drug administration. More importantly, the assay used to assess salivary oxytocin was not sensitive enough, and many of the samples were below detectable limits. Even within these constraints, however, MDMA, and to a lesser extent MA, increased oxytocin levels. Interestingly, oxytocin levels after MDMA were related to feeling close to the partner, whereas oxytocin levels after MA were not. The robust increase in oxytocin after MDMA is consistent with other studies^10,42^, and lesser effect of MA is consistent with previous reports that MA has little effect on oxytocin levels^21,26^. The correlation between oxytocin levels and ratings of partner closeness after MDMA is consistent with the idea that feelings of closeness may be partly influenced by its serotonin-mediated effects on oxytocin^20^. The lack of correlation between oxytocin and closeness after MA suggests that other actions of this drug (e.g., dopaminergic and noradrenergic), may mediate the closeness effect^43^. Interestingly, MA, but not MDMA, increased participants’ reports of attentiveness during the interaction and decreased their ratings of negative aspects of the conversation (e.g., difficulty in communication, and feeling drained of energy from the interaction). These results might reflect the relatively stronger effect of MA, compared to MDMA, on dopamine function. Future pharmacological studies with specific receptor agonists or antagonists agents may elucidate the contributions of various monoaminergic neurotransmitters for each drug.

The results of this study have implications for MDMA-assisted therapy. First, they raise the possibility that some of the therapeutic effects of MDMA are related to enhanced feelings of connectedness between the patient and therapist. This feeling of connectedness could help patients feel safe and trusting, thereby facilitating deeper emotional exploration. Second, the construct of connectedness may be of value in designing MDMA-assisted therapy protocols, providing a measurable target to assess patient-therapist interactions. Third, the findings with MA raise the interesting question of whether drugs other than MDMA might also facilitate psychotherapy, by affecting the quality of the patient-therapist connection. More broadly, understanding the behavioral processes by which MDMA enhances social interactions is important to help therapists optimize the beneficial effects of the drug.

This project had limitations. First, the two studies were not directly comparable. Only a single dose of each drug was tested, making it difficult to compare across drugs. Further, all participants in Study 1 had used MDMA at least once in their lifetime whereas few of the participants in Study 2 reported prior MDMA use. It is unlikely that the prior drug use history contributed to responses in these two studies, as we have shown previously^44^ that history of prior MDMA has little effect on acute responses to the drug. We are not aware of studies examining the effects of MDMA or other stimulant drugs on acute responses to MA. Nevertheless, it is not known whether this difference in prior drug use contributed to the present findings. Another major limitation is that many of the saliva samples from placebo sessions had undetectable levels of oxytocin, and for these samples the minimum threshold value was used to calculate oxytocin levels. Therefore, our findings are considered preliminary, and the magnitude of salivary oxytocin detected may not reflect true physiological values. Future studies using more sensitive methods for measuring oxytocin levels are needed. Another limitation is that saliva samples for oxytocin detection were only collected once post-capsule administration without a baseline measurement, while it is known that baseline oxytocin concentrations vary from day to day^45^. Finally, we used several validated measures of connection, but other measures of this construct, including scales assessing broader dimensions of connectedness to self and to the world^46^ may also be useful for future studies.

In conclusion, the present study found that, relative to placebo, both MDMA and MA promoted feelings of connectedness to strangers during controlled social interactions. Although this effect was expected for MDMA, it was surprising that MA produced similar effects. MDMA is known for its prosocial effects (e.g., at rave parties, and facilitating interactions with therapists), whereas MA is known mainly as a treatment for ADHD and obesity, and for its abuse liability. Yet, in the present study, when participants were tested in the same social setting, the drugs produced similar effects. The unexpected finding that MA also increased feelings of closeness raises the novel possibility that this drug also might have potential as an adjunct to psychiatric treatment. An important future direction will be to investigate how social contexts alter responses to these drugs. The procedure used here offers a good model to study such effects.

### Supplementary Information

Supplementary Tables.

## Data Availability

The datasets generated during and/or analyzed during the current study are available from the corresponding author on reasonable request.
