# Supplementary material for: Drug-induced social connection: both MDMA and methamphetamine increase feelings of connectedness during controlled dyadic conversations
Source: Sci Rep. 2023 Sep 22;13:15846. doi: 10.1038/s41598-023-43156-0 (PMC10516994; doi:10.1038/s41598-023-43156-0)
Supplement: Supplementary file 1 — Supplementary Tables. [file 41598_2023_43156_MOESM1_ESM.doc]

**Supplementary Materials**

**Supplementary Table 1.** Mean CDCS subscale ratings (Studies 1 [MDMA] and 2 [MA]).

|  | **MDMA Study (*N*=17)** | | | **MA Study (*N*=19)** | | |
| --- | --- | --- | --- | --- | --- | --- |
| ***Connection During Conversations Scale*** | PLAC  Mean  ± SEM | MDMA  Mean  ± SEM | *P* | PLAC  Mean  ± SEM | MA  Mean  ± SEM | *P* |
|  |  |  |  |  |  |  |
| Shared Reality | 5.2 ± 0.4 | 5.6 ± 0.2 | 0.3 | 5.0 ± 0.2 | 5.8 ± 0.2 | 0.02* |
| Partner Responsiveness | 4.6 ± 0.3 | 5.5 ± 0.2 | 0.002** | 5.2 ± 0.2 | 5.7 ± 0.2 | 0.02* |
| Participant Interest | 5.1 ± 0.3 | 6.1 ± 0.2 | 0.04* | 5.6 ± 0.3 | 6.7 ± 0.1 | 0.001** |
| Affective Experience | 5.6 ± 0.2 | 5.9 ± 0.3 | 0.5 | 5.2 ± 0.3 | 6.6 ± 0.1 | 0.001** |

**Supplementary Table 2.** Peak change from baseline mean subjective ratings during each in-lab session (Studies 1 [MDMA] and 2 [MA]).

|  | **MDMA Study (*N*=17)** | |  | **MA Study (*N*=19)** | |  |
| --- | --- | --- | --- | --- | --- | --- |
| **DEQ** | PLAC ± SEM | MDMA ± SEM | ***P*** | PLAC ± SEM | MA ± SEM | ***P*** |
| *Feel* | 30.9 ± 6.9 | 86.1 ± 3.9 | <0.001*** | 22.8 ± 4.9 | 53.7 ± 5.4 | <0.001*** |
| *Like drug* | 39.9 ± 8.9 | 91.2 ± 3.1 | <0.001*** | 28.7 ± 6.1 | 69.8 ± 6.7 | <0.001*** |
| *Dislike drug* | 23.2 ± 7.4 | 47.1 ± 6.0 | 0.02* | 15.6 ± 4.3 | 25.6 ± 5.3 | 0.02* |
| *High* | 29.5 ± 7.0 | 87.5 ± 3.0 | <0.001*** | 17.1 ± 4.1 | 45.6 ± 5.6 | <0.001*** |
| *Want more* | 42.6 ± 8.4 | 71.1 ± 6.6 | 0.01* | 29.6 ± 6.5 | 62.1 ± 6.9 | <0.001*** |
| **VAS** |  |  |  |  |  |  |
| *Anxious* | -10.2 ± 7.1 | 2.0 ± 9.8 | 0.3 | -11.7 ± 6.6 | -9.2 ± 8.1 | 0.8 |
| *Stimulated* | 7.7 ± 9.3 | 46.1 ± 7.8 | 0.003** | 6.9 ± 8.2 | 33.9 ± 5.5 | 0.01* |
| *Insightful* | -1.8 ± 8.2 | 40.1 ± 8.2 | <0.001*** | 9.2 ± 7.1 | 31.6 ± 6.3 | 0.04* |
| *Sociable* | -3.5 ± 8.2 | 32.0 ± 8.9 | 0.005* | 3.9 ± 6.6 | 27.4 ± 6.2 | 0.03* |
| *Confident* | 4.1 ± 4.8 | 19.6 ± 8.8 | 0.08 | 3.4 ± 5.6 | 13.3 ± 5.2 | 0.3 |
| *Lonely* | -9.1 ± 5.9 | -1.9 ± 6.8 | 0.5 | 2.3 ± 5.5 | -5.6 ± 6.8 | 0.3 |
| *Playful* | 15.1 ± 6.6 | 35.0 ± 8.9 | 0.1 | 10.6 ± 7.2 | 30.6 ± 5.9 | 0.08 |
| *Loving* | -2.7 ± 8.0 | 33.4 ± 8.5 | <0.001*** | 0.1 ± 5.1 | 26.7 ± 4.9 | 0.001** |
| *Friendly* | 4.8 ± 5.9 | 30.9 ± 7.0 | 0.002** | -5.8 ± 6.1 | 23.1 ± 5.5 | 0.01* |
| *Restless* | 15.2 ± 9.0 | 27.4 ± 12.1 | 0.4 | -2.4 ± 7.6 | -3.3 ± 10.0 | 1.0 |
| *Trusting* | 0.2 ± 7.0 | 24.9 ± 6.8 | 0.008* | 1.5 ± 6.2 | 10.0 ± 5.7 | 0.3 |
| *Appreciated* | -0.9 ± 7.7 | 33.6 ± 5.9 | <0.001*** | 5.6 ± 5.3 | 13.3 ± 5.0 | 0.3 |
| *Grateful* | -0.8 ± 6.9 | 24.2 ± 8.9 | 0.01* | 2.8 ± 3.9 | 8.8 ± 4.7 | 0.4 |
| *Understood* | 1.2 ± 6.1 | 19.3 ± 8.4 | 0.08 | 3.3 ± 5.3 | 18.8 ± 4.5 | 0.05* |
| *Loved* | -5.0 ± 5.7 | 30.4 ± 6.5 | <0.001*** | -0.6 ± 4.0 | 6.9 ± 4.3 | 0.2 |
| **POMS** |  |  |  |  |  |  |
| *Anger* | -1.3 ± 0.7 | -1.2 ± 1.0 | 1.0 | 0.8 ± 0.4 | 0.0 ± 0.4 | 0.1 |
| *Anxiety* | -2.1 ± 1.2 | 2.5 ± 1.9 | 0.01* | -2.5 ± 1.3 | -0.7 ± 1.0 | 0.4 |
| *Confusion* | -0.2 ± 1.0 | 2.9 ± 1.0 | 0.003** | -0.1 ± 0.4 | -1.1 ± 0.7 | 0.2 |
| *Depression* | -2.5 ± 1.2 | -2.0 ± 1.6 | 0.7 | -0.3 ± 0.2 | -0.9 ± 0.7 | 0.3 |
| *Elation* | -1.1 ± 1.1 | 8.8 ± 2.2 | 0.001** | -0.7 ± 1.0 | 3.1 ± 1.1 | 0.004** |
| *Fatigue* | -2.1 ± 1.3 | -0.2 ± 1.8 | 0.4 | 0.1 ± 0.9 | -2.5 ± 0.8 | 0.05* |
| *Friendliness* | -1.5 ± 1.6 | 7.2 ± 2.6 | 0.01* | -2.2 ± 1.1 | 3.8 ± 1.5 | 0.002** |
| *Vigor* | 0.3 ± 1.6 | 12.6 ± 2.2 | <0.001*** | -0.3 ± 1.0 | 6.4 ± 1.6 | 0.005** |

**Supplementary Table 3.** Peak change from baseline cardiovascular measures and mean salivary oxytocin levels (Studies 1 [MDMA] and 2 [MA]).

|  | **MDMA Study (*N*=17)** | | | **MA Study (*N*=19)** | | |
| --- | --- | --- | --- | --- | --- | --- |
|  | PLAC  Mean ± SEM | MDMA  Mean ± SEM | *P* | PLAC  Mean ± SEM | MA  Mean ± SEM | *P* |
| Systolic BP | -6.4 ± 4.5 | 21.1 ± 5.8 | <0.001*** | -2.0 ± 3.2 | 14.6 ± 4.2 | 0.005** |
| Diastolic BP | -1.6 ± 3.6 | 14.2 ± 4.1 | 0.009* | -2.5 ± 2.6 | 10.3 ± 2.9 | 0.001** |
| Heart rate | 7.3 ± 3.4 | 40.2 ± 4.5 | <0.001*** | 4.0 ± 3.7 | 16.4 ± 4.2 | 0.05* |
| Oxytocin levels (pcg/mL) | 0.3 ± 0.02 | 1.4 ± 0.3 | 0.001** | 0.3 ± 0.001 | 0.8 ± 0.2 | 0.02* |
|  |  |  |  |  |  |  |
